# *Bathynomus propinquus* Richardson, 1910, a valid species of supergiant deep-sea isopod from the Philippines, with notes on *B. wilsoni* Ahyong, 2025 (Crustacea, Isopoda, Cirolanidae)

**DOI:** 10.3897/zookeys.1286.200843

**Published:** 2026-07-23

**Authors:** Shane T. Ahyong, Conni M. Sidabalok, Chien-Hui Yang, Peter K. L. Ng

**Affiliations:** 1 Australian Museum Research Institute, Sydney, NSW 2010, Australia, and School of Biological, Earth & Environmental Sciences, University of New South Wales, Kensington, NSW 2052, Australia Lee Kong Chian Natural History Museum (LKCNHM), 2 Conservatory Drive, National University of Singapore Singapore Singapore; 2 Research Center for Biosystematics and Evolution, National Research and Innovation Agency (BRIN), KST Soekarno, Jalan Raya Jakarta Bogor Km 46, Cibinong 16911, Indonesia Institute of Marine Biology and Center of Excellence for the Oceans, National Taiwan Ocean University Keelung Taiwan; 3 Institute of Marine Biology and Center of Excellence for the Oceans, National Taiwan Ocean University, Keelung 202301, R.O.C, Taiwan Australian Museum Research Institute, Sydney, NSW 2010, Australia, and School of Biological, Earth & Environmental Sciences, University of New South Wales Kensington Australia; 4 Lee Kong Chian Natural History Museum (LKCNHM), 2 Conservatory Drive, National University of Singapore, Singapore 117377, Singapore Research Center for Biosystematics and Evolution, National Research and Innovation Agency (BRIN) Cibinong Indonesia

**Keywords:** *
Bathynomus
*, Cirolanidae, Crustacea, deep-sea, giant isopod, Isopoda, Philippines

## Abstract

The deep-sea isopod *Bathynomus propinquus* Richardson, 1910, was described based on a single male juvenile from the Verde Island Passage, central-western Philippines, but owing to its immaturity, had long been considered unidentifiable and treated as a nomen dubium. Settling the identity of *B. propinquus*, however, is important because the name remains available and nomenclaturally precedes all other species of supergiant *Bathynomus* in the Indo-West Pacific, with its type locality within the reported range of *B. jamesi* Kou, Chen & Li in Kou et al. 2017, raising the obvious possibility that the two nominal species could be conspecific. Detailed studies of *B. jamesi* sensu lato in the South China Sea revealed that it comprises two species distinguished by morphology and strong mitochondrial sequence divergence: a northern form (*B. jamesi* sensu stricto) ranging from Vietnam to off Hainan and the Dongsha Islands, and a southeastern form (here identified as *B. propinquus*) from the Verde Island Passage, southern Luzon, Philippines. *Bathynomus propinquus* is here considered a valid species based on re-evaluation of the holotype, additional material, new characters, size-related variation, and molecular data. *Bathynomus propinquus* belongs to the group of Indo-West Pacific supergiants sharing upturned pleotelson spines, most of which were formerly confused with *B. kensleyi* Lowry & Dempsey, 2006 from the Coral Sea, herein referred to as the *B. kensleyi* group. Given the new data on supergiants in the South China Sea, a key to the species of the *B. kensleyi* group is provided. A new record of *B. wilsoni* Ahyong, 2025 from the northern Sulu Sea is also reported.

## Introduction

The deep-water isopod genus *Bathynomus* A. Milne-Edwards, 1879 is best known for its type species, *B. giganteus* A. Milne-Edwards, 1879, described from the Gulf of Mexico, with ongoing current discovery of new species (e.g., Sidabalok et al. 2020; Huang et al. 2022; Ahyong 2025; Ng et al. 2025; Riehl et al. 2026). *Bathynomus giganteus*, the largest of the supergiant species in the genus is known to reach at least 450 mm body length (with an unconfirmed report at 500 mm; Lowry and Dempsey 2006: 166). Following its discovery, *B. giganteus* was also reported from the Indo-West Pacific including the Arabian Sea and Bay of Bengal (Lloyd 1908; Annandale 1909; Holthuis and Mikulka 1972) as well as localities in East and Southeast Asia, primarily in the South China Sea (White 1986; Soong 1992), affording *B. giganteus* a supposedly widespread, almost global, distribution. Revision of the Indo-Pacific species of *Bathynomus*, however, showed that *B. giganteus* is restricted to the western Atlantic and that all Indo-West Pacific records are based on other species (Lowry and Dempsey 2006).

Lowry and Dempsey (2006) referred previous records of *B. giganteus* from the South China Sea (Philippines, Taiwan) to a new species, *B. kensleyi* Lowry & Dempsey, 2006, with its type locality in the Coral Sea off northeastern Australia, suggesting a strongly disjunct anti-equatorial distribution. In the northern range of *B. kensleyi*, Lowry and Dempsey (2006) included specimens from the northern South China Sea and off the central-western Philippines, as well as specimens from the Sulu Sea, southern Philippines. In recent years, close taxonomic scrutiny of the supergiants from the South China Sea and adjacent localities (Kou et al. 2017; Huang et al. 2022; Huang and Bruce 2024; Ahyong 2025; Huang and Kawai 2025; Ng et al. 2025, 2026) have recognised several *B. kensleyi*-like species in the region, all sharing upcurved posterior pleotelson spines (we note that another species having upcurved pleotelson spines also occurs in the Andaman Sea, *B. lowryi* Bruce & Bussarawit, 2004). The seemingly disjunct populations of *Bathynomus kensleyi* reported by Lowry and Dempsey (2006) actually represent different species, with *B. kensleyi* sensu stricto occurring only south of the equator in the Coral Sea off northeastern Australia (see Huang and Bruce 2024). North of the equator, previous records of *B. kensleyi* were referred to two species, *B. jamesi* Kou, Kou, Chen & Li in Kou et al. 2017 (South China Sea from off Vietnam, Hainan, Dongsha, and the central-western Philippines) (Huang et al. 2022; Huang and Bruce 2024; Ng et al. 2025) and *B. wilsoni* Ahyong, 2025 (Sulu Sea; Ahyong 2025). *Bathynomus jamesi* was originally described based on juveniles but Huang et al. (2022) linked the juvenile type material of *B. jamesi* (from off Hainan) to adults from Dongsha based on COI sequences, and Ng et al. (2025) documented the species from off Vietnam based on new adult material. Ng et al. (2025) also identified Lowry and Dempsey’s (2006) record of *B. kensleyi* from off western Luzon, Philippines, as *B. jamesi*.

Two other species of *B. kensleyi*-like supergiants were recently described from the South China Sea: *B. vaderi* Ng, Sidabalok & Nguyen, 2025 and *B. paracelensis* Huang & Kawai, 2025, with the latter demonstrated to be a junior synonym of the former (Ng et al. 2026). In addition to the four aforementioned species, a fifth supergiant has actually been recorded from the South China Sea: the long-unresolved *B. propinquus* Richardson, 1910.

*Bathynomus propinquus* was described based on a single male juvenile from the central Philippines (Richardson 1910). It is a species of supergiant, but owing to its immaturity, the identity of *B. propinquus* has remained uncertain, although it has been considered nearest to *B. giganteus* (Richardson 1910; Shih 1972; Monod 1973; Cocke 1987; Soong 1992). In revising the Indo-West Pacific species of *Bathynomus*, Lowry and Dempsey (2006) considered *B. propinquus* to be unidentifiable and treated it as a nomen dubium; since then, it has not been considered in studies of the genus. The name, however, remains available, and settling the identity of *B. propinquus* is important because it nomenclaturally precedes all other supergiants in the Indo-West Pacific and because its type locality is within the reported range of *B. jamesi*, and adjacent to that of *B. vaderi* and *B. wilsoni*, raising the obvious possibility that it may be a senior synonym of one of these species. We herein reevaluate the identity of *B. propinquus* based on interpretation of the morphology of the holotype in the light of new characters, size-related variation in related species, additional material, and molecular data.

## Materials and methods

Specimens examined are deposited in the collections of the Australian Museum, Sydney (**AM**); Zoological Reference Collection of the Lee Kong Chian Natural History Museum, National University of Singapore (**ZRC**); Muséum national d’Histoire naturelle, Paris (**MNHN**); Museum Zoologicum Bogoriense, BRIN, Cibinong, Indonesia (**MZB**); Museum and Art Gallery of the Northern Territory, Darwin (**NTM**); Queensland Museum Kurilpa (**QM**); and National Museum of Natural History, Smithsonian Institution, Washington D.C. (**USNM**). Specimen measurements, in millimetres (mm) are of the maximum total length, measured longitudinally from the apex of the clypeus to the apex of the central pleotelson spine. Morphological terminology follows Ahyong (2025) and Ng et al. (2026). In the ‘Description’ of *B. propinquus*, morphometric and meristic values for the holotype are given in square brackets where they differ from those of the adult specimen.

New mitochondrial sequences from *B. wilsoni* (ZRC 2018.1076; COI, GenBank accession number PZ567722) and *B. raksasa* Sidabalok, Wong & Ng, 2020 (ZRC 2020.0015; GenBank accession number PZ567723 (COI); PZ568892 (16S)) were generated following the protocols outlined by Ng et al. (2026). The new sequences were combined with the *Bathynomus* dataset of Ng et al. (2026) and analysed under the same parameters. Except for sequences of *B. wilsoni* and *B. raksasa* newly generated herein, see Ng et al. (2026) for GenBank accession numbers and specimen voucher details. Corrected pairwise distances were calculated based on the Kimura 2-parameter model (K2P; Kimura 1980) in MEGA v. 11 (Tamura et al. 2021). A maximum-likelihood (ML) tree was constructed using the IQ-TREE v. 3.0.1 (Wong et al. 2025) based on the TIM2+G4+F model and with *Excirolana hirsuticauda* Menzies, 1962 as the outgroup; topological robustness was assessed with 1,000 bootstrap replicates.

### Comparative material

*Bathynomus jamesi* Kou, Chen & Li, 2017: ZRC 2024.0118, 3 males (335–419 mm), 1 female (320 mm), South China Sea off Quảng Ngãi, Khánh Hòa and/or Phú Yên Provinces, central Vietnam, trawled, coll. Thanh Son Nguyen, April 2024; ZRC 2024.0179, 1 male (410 mm), same data as preceding; AM P109291, 1 male (395 mm), same data as preceding; ZRC, 2 males (371 mm, 400 mm), 2 females (288 mm, 314 mm), Dongsha, South China Sea, bottom trawl, from fishermen, C.-H. Yang, 2023; AM P111577, 1 female (284 mm), South China Sea, off Ly Son Island, Quảng Ngãi Province, Vietnam, commercial trawler, coll. Nguyễn Phúc Cẩm Tú, April 2026; AM P111578, 1 male (288 mm), same data as preceding.

*Bathynomus kensleyi* Lowry & Dempsey, 2006: NTM Cr3425, male holotype (277 mm), southeast of Swain Reefs, Coral Sea, 22°55.1'S, 154°21.25'E, 590–606 m, 17 November 1985; AM P68557, 1 female (252 mm), east of Flynn Reef, Coral Sea, Queensland, Australia, 16°37.82'S, 146°23.08'E, 1000 m, baited trap, stn QLD 950, RV Sunbird, coll. J.K. Lowry, P. Freewater & W. Vader, 7–8 June 1993; AM P68686, 1 male (242 mm), east of Flynn Reef, Coral Sea, Queensland, Australia, 16°37.82'S, 146°23.08'E, 1000 m, baited trap, stn QLD 932, RV Sunbird, coll. J.K. Lowry, P. Freewater & W. Vader, RV Sunbird, 6–7 June 1993; AM P68689, 1 juvenile male (69 mm), 3 mancas (43–47 mm), same, stn QLD 932; AM P68690, 5 mancas (44–51 mm), east of Flynn Reef, Coral Sea, Queensland, Australia, 16°37.82'S, 146°23.08'E, 1000 m, baited trap, stn QLD 931, RV Sunbird, coll. J.K. Lowry, P. Freewater & W. Vader, RV Sunbird, 6 June 1993; QM W28011, 1 male (126 mm), northwest of Lihou Reef, Coral Sea, 16°55'S, 151°34'E, 880 m, trawled, RV Soela CR6 sta 78, coll. P. Davie, 6 October 1985; QM W29630, 1 male (315 mm), east of Heron Island, 23°15'11.4"S, 153°52'18.6"E, coll. D. Hand, November 2022.

*Bathynomus raksasa* Sidabalok, Wong & Ng, 2020: ZRC 2020.0015, female paratype (298 mm), east of Tinjil Island, Indonesia, 6°59.778'S, 105°55.224'E, 957 m, beam trawl, SJADES sta. CP 28, 28 March 2018.

*Bathynomus vaderi* Ng, Sidabalok & Nguyen, 2025: ZRC 2022.0621, male holotype (279 mm), ca. 50 nautical miles off Quy Nhơn City, Bình Định Province, south-central Vietnam, from deep-water (depth not recorded), trawled, 27 March 2022; ZRC 2024.0176, male paratype (270 mm), offshore of Quy Nhơn province, south-central Vietnam, ca. 50 nautical miles from shore, 27 March 2022; ZRC 2024.0180, 2 male paratypes (305 mm, 337 mm), off Quảng Ngãi, Bình Định, Khánh Hòa and/or Phú Yên Provinces, central Vietnam, trawled, September 2024.

## Results

### The holotype of *Bathynomus propinquus* Richardson, 1910

At the time of its description, *B. propinquus* was compared with *B. giganteus*, the only other congener then known (Richardson 1910). Subsequent workers followed Richardson (1910) in their comparisons and considerations of *B. propinquus* (e.g. Shih 1972; Monod 1973; Cocke 1987; Soong 1992).

**Figure 1.**
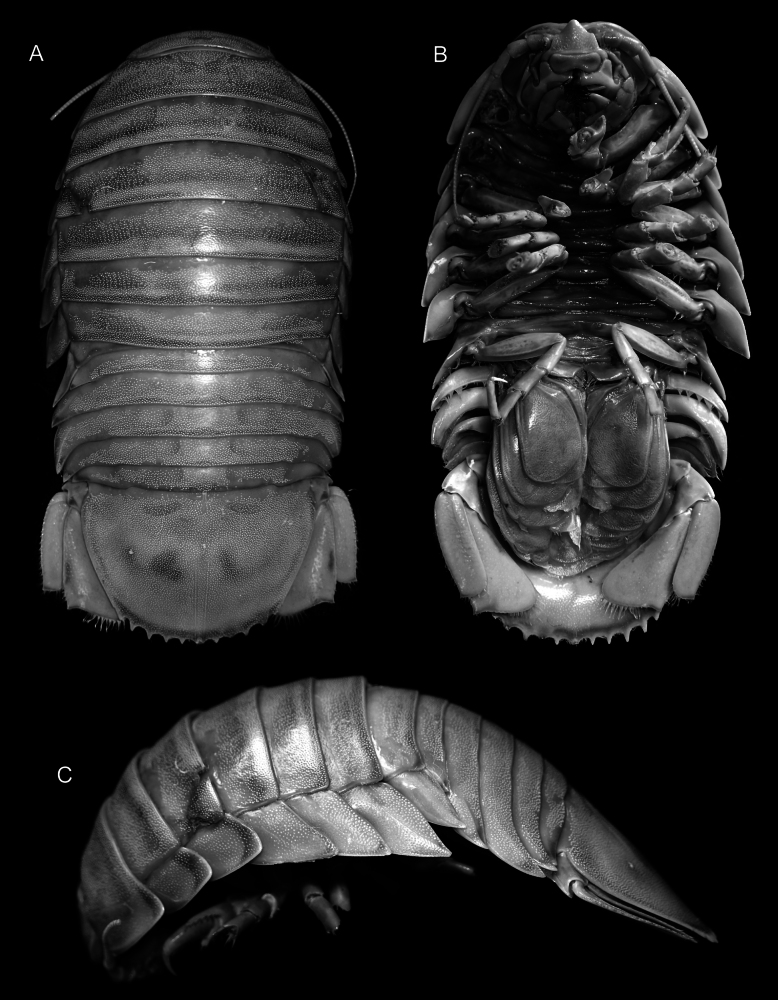
*Bathynomus propinquus* Richardson, 1910, holotype juvenile male (88 mm), Verde Island Passage, Philippines, USNM 40909: **A**. Dorsal habitus; **B**. Ventral habitus; **C**. Left lateral habitus.

**Figure 2.**
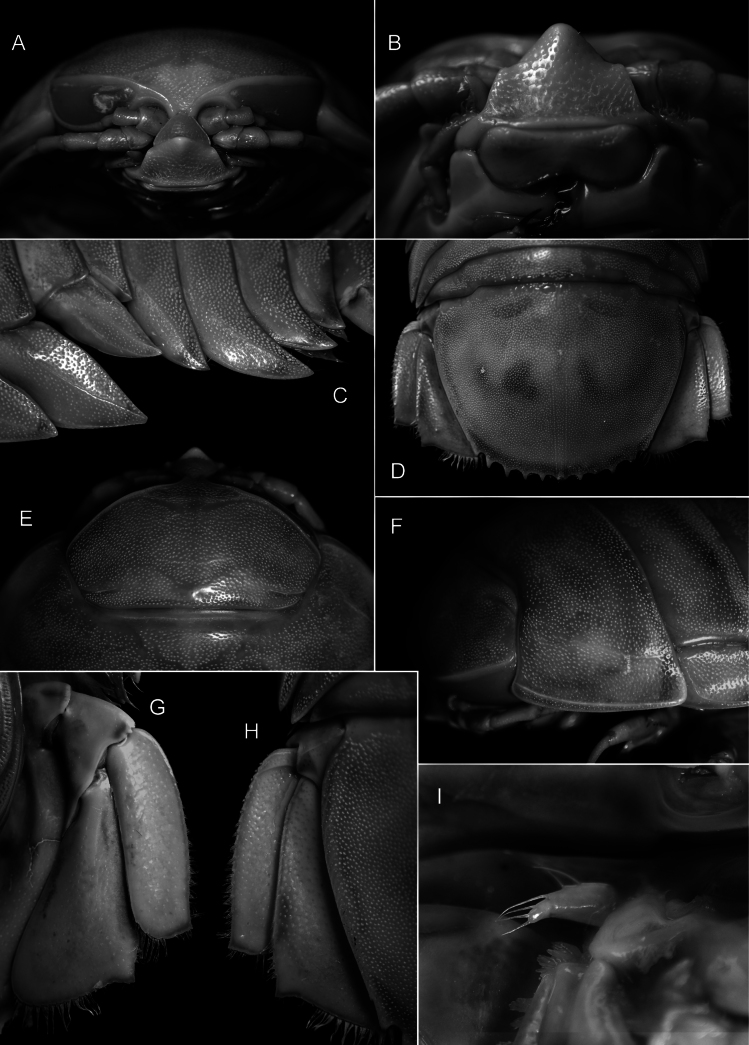
*Bathynomus propinquus* Richardson, 1910, holotype juvenile male (88 mm), Verde Island Passage, Philippines, USNM 40909: **A**. Cephalon, anterior view; **B**. Clypeal region; **C**. Pereonites 6–7 and pleonites 1–5, left lateral view; **D**. Pleotelson and uropods, dorsal view; **E**. Cephalon, dorsal view; **F**. Cephalon and pereonite 1, left lateral view; **G, H**. Left uropod, ventral view and dorsal view, respectively; **I**. Right pleopod 5 exite.

Richardson (1910) distinguished *B. propinquus* from *B. giganteus* based on several features including the length of the antennal flagellum, the shape of the frontal lamina, carination of the pereonal coxal plates (incorrectly referred to as epimera by Richardson 1910: 4), and aspects of the uropods, the most important being the slenderness of the exopod. Each of these seemingly distinctive features listed by Richardson (1910) are subject to allometric change, and although their condition in adults can be diagnostic, they are taxonomically ineffective in juveniles. The holotype of *B. propinquus* (Figs 1, 2) is in poor condition, including damaged apices of the pleotelson spines, but overall is still sufficiently intact for taxonomic evaluation. The specimen is a juvenile male with erupted penes and a well-developed pereopod 7 (Fig. 1B), but the ‘immature’ condition of many characters diagnostic in adults is evident. In particular, the lateral margins of the clypeus (Fig. 2B) are strongly divergent in mancas and early juveniles, and do not become parallel-subparallel until late juvenile-subadult stages. Similarly, the antennal flagellum becomes proportionally shorter with increasing body size. The pleotelson spines are dorsoventrally flattened (Figs 1A, 1C, 2D) and are yet to attain the slender proportions and ovate-circular cross-section of late-juveniles and adults (Figs 4A, 4C, 5F–H). Most workers have focused on the slenderness of uropodal exopod (Fig. 2G, H) highlighted by Richardson (1910) as a key distinguishing feature of *B. propinquus*, but this feature, which is diagnostic in adults, is subject to allometry and so cannot be used in direct comparison between juveniles and adults. Lowry and Dempsey (2006) examined the holotype of *B. propinquus* and considered it to be taxonomically uninterpretable, declaring the species to be a nomen dubium.

**Figure 3.**
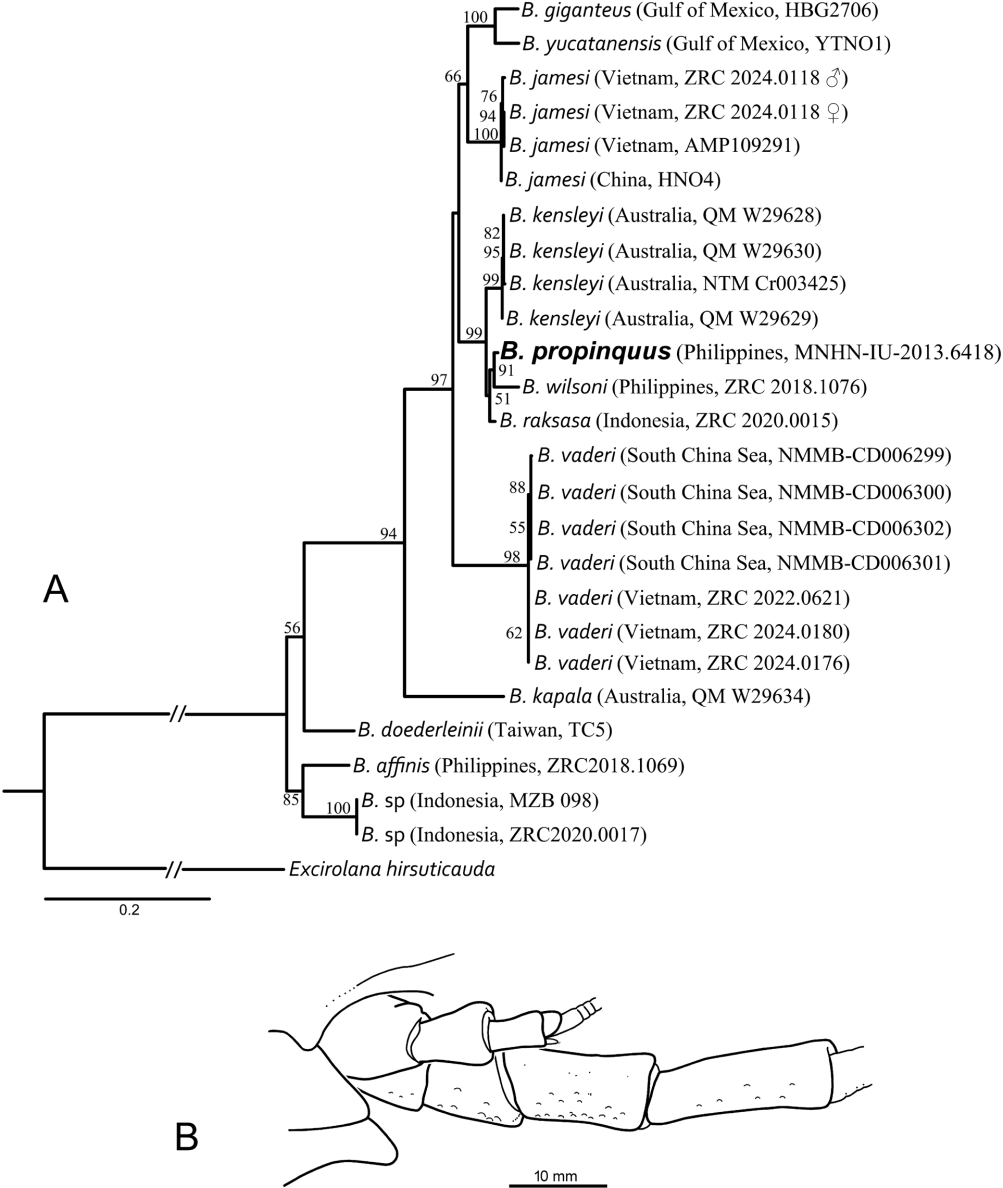
**A**. Maximum-likelihood topology (TIM2+G4+F model) of species of *Bathynomus* based on combined sequences of mitochondrial COI (657 bp) and 16S rRNA (540 bp) genes, rooted to *Excirolana hirsuticauda* Menzies, 1962; geographic origin, museum and voucher number indicated in brackets. Bootstrap support exceeding 50% indicated at nodes. Except for sequences of *B. wilsoni* and *B. raksasa* newly generated herein, see Ng et al. (2026) for GenBank accession numbers and specimen voucher details. **B**. *Bathynomus propinquus* Richardson, 1910, male (320 mm), Verde Island Passage, Philippines, MNHN-IU-2013.6418 (IS.2290), left antenna and antennule.

**Figure 4.**
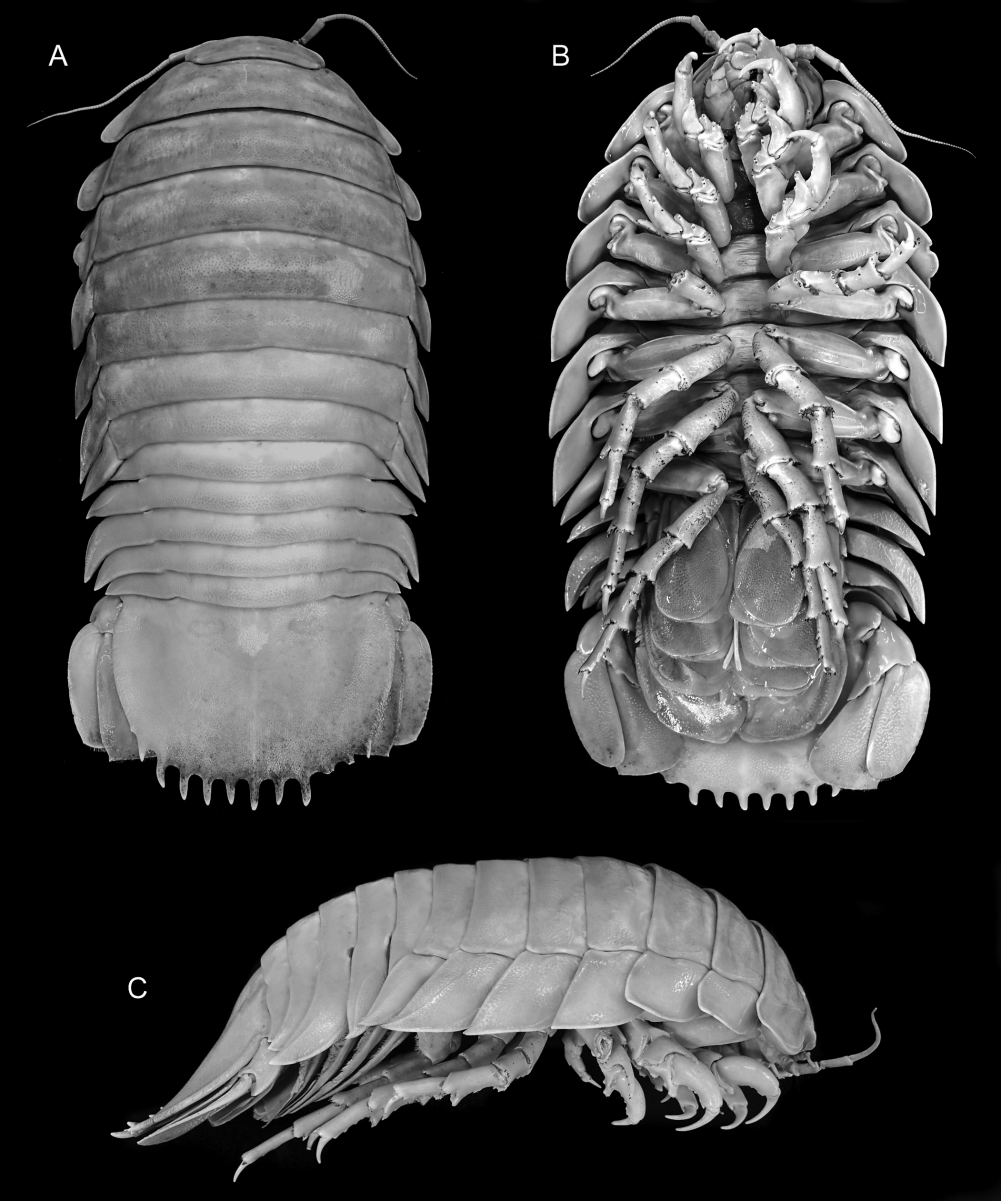
*Bathynomus propinquus* Richardson, 1910, male (320 mm), Verde Island Passage, Philippines, MNHN-IU-2013.6418 (IS.2290): **A**. Dorsal habitus; **B**. Ventral habitus; **C**. Right lateral habitus.

**Figure 5.**
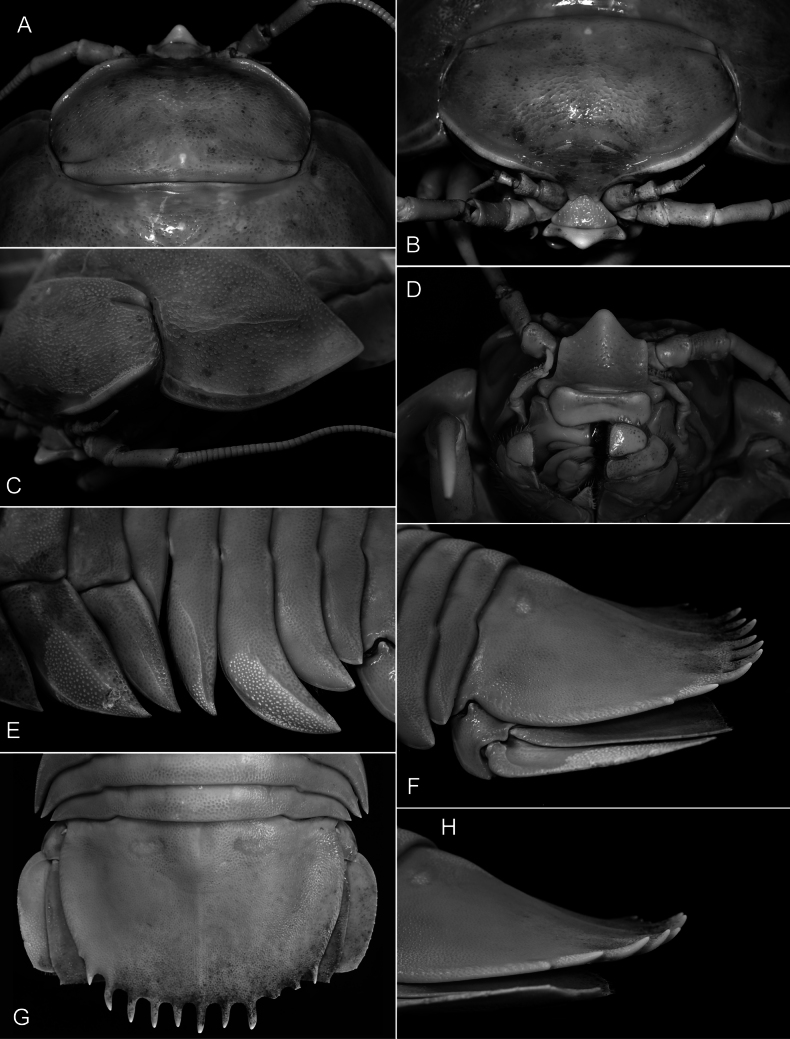
*Bathynomus propinquus* Richardson, 1910, male (320 mm), Verde Island Passage, Philippines, MNHN-IU-2013.6418 (IS.2290): **A**. Cephalon, dorsal view; **B**. Cephalon, oblique anterior view; **C**. Cephalon and pereonite 1, left lateral view; **D**. Clypeal region and oral field; **E**. Pereonites 6–7 and pleonites 1–5, left lateral view; **F**. Posterior pleonites and pleotelson, left lateral view; **G**. Pleotelson and uropods, dorsal view; **H**. Pleotelson, left lateral view.

*Bathynomus propinquus* has been widely mentioned in the literature as citations of the original description. The only other published specimen record of *B. propinquus* apart from the holotype, that of Monod (1973: figs 54, 55) from New Caledonia, is referable to *B. richeri* Lowry & Dempsey, 2006, as is evident from Monod’s figures. Thus, until now, *B. propinquus* remained known only from the Philippine holotype.

### The identity of *Bathynomus propinquus* Richardson, 1910

Our reassessment of supergiants in the South China Sea based on morphological and molecular data shows that *B. jamesi* sensu Ng et al. (2025, 2026) and Ahyong (2025) comprises two species: a northern form occurring on the continental slope in an arc ranging from Vietnam to off Hainan and the Dongsha Islands, and a southeastern form, also from slope depths, from the Verde Island Passage, southern Luzon, Philippines. The northern form corresponds to *B. jamesi* sensu stricto and the southeastern form is a separate species. The molecular separation between the two forms is not only supported by strong divergence in COI (10.8–11.2%) and 16S (5.7–6.4%) (Ng et al. 2026, as *B. cf. jamesi*) but also by disparate phylogenetic position, with the southeastern form being sister to *B. wilsoni* from the Sulu Sea, rather than *B. jamesi*. Notably, the sequenced specimen of the southeastern form examined here (MNHN-IU-2013.6418) was collected from Verde Island Passage less than 20 km north of the type locality of *B. propinquus*. This raises the question over whether the juvenile holotype of *B. propinquus* is identifiable with adults of one of the two *B. jamesi*-like forms (whether *B. jamesi* sensu stricto or the southeastern form, referred to as *B. cf. jamesi* by Ng et al. 2026), or even *B. wilsoni* or *B. vaderi*, which are known from adjacent localities. Our comparisons show that the juvenile holotype of *B. propinquus* and adults of other regional supergiant species now recognised clearly differ in the shape of the clypeus, orientation of the pleotelson spines, the slenderness of the uropodal exopod, and length of the antennal peduncle, features subject to allometric change, confounding Lowry and Dempsey’s (2006) taxonomic efforts with the species. We have, however, identified additional diagnostic characters in *Bathynomus*, particularly as determined from species closely related to (and previously misidentified with) *B. kensleyi*, namely *B. jamesi* and *B. wilsoni*, in addition to *B. vaderi* (see Huang et al. 2022; Ahyong 2025; Ng et al. 2025, 2026; this study). Despite the immaturity of the holotype of *B. propinquus*, our study of a good series of specimens of related supergiants, including juveniles, revealed other ontogenetically stable characters that can reliably link juveniles with adults. The juvenile holotype of *B. propinquus* and adult of the southeastern form share similarly short lateral cephalic incisions (length about 0.2 × distance between incisions; Figs 2E, 5A, 5B) (≥ 0.3 in *B. jamesi*, *B. vaderi* and *B. wilsoni*; Ahyong 2025: fig. 8A, C, D) and similar alignment of the pleonite 3–5 pleura (= epimera) in which the pleuron 3 apex does not reach that of pleuron 4 and neither reach to the end of pleuron 5 (a feature also of *B. jamesi* and *B. vaderi*, but not *B. wilsoni* or *B. kensleyi* from Australia; Fig. 7). However, a hitherto unused morphological character is also effective here: the anterolateral carination of pereonite 2 in the way the upper marginal carina meets the anterior margin of the somite. In *B. kensleyi* and *B. jamesi*, the upper marginal carina of pereonite 2 merges with the anterior margin in an even curve (Fig. 7B, D), but meets at a slightly obtuse, but nevertheless distinct, angle in the southeastern form, *B. wilsoni* and in *B. vaderi* (Fig. 7A, C, E). The combination of this angular junction between the upper marginal carina and anterior margin of pereonite 2, the short lateral cephalic incisions (length about 0.2 × distance between incisions) and similar alignment of the pleonite 3–5 pleura is only found in the southeastern form and the juvenile holotype of *B. propinquus* (Figs 2F, 5C, 7A). That they were also collected in close geographic proximity from the Verde Island Passage, and that no other species of *Bathynomus* is known from the area, provides strong evidence that the examined specimens represent the respective juvenile and adult forms of *B. propinquus*. The *B. cf. jamesi* of Ng et al. (2026) is now reidentified as *B. propinquus*. We thus conclude on the basis of morphological and molecular evidence that *B. propinquus* is a valid species separate from other named species; it is re-described below based on adult features.

**Figure 6.**
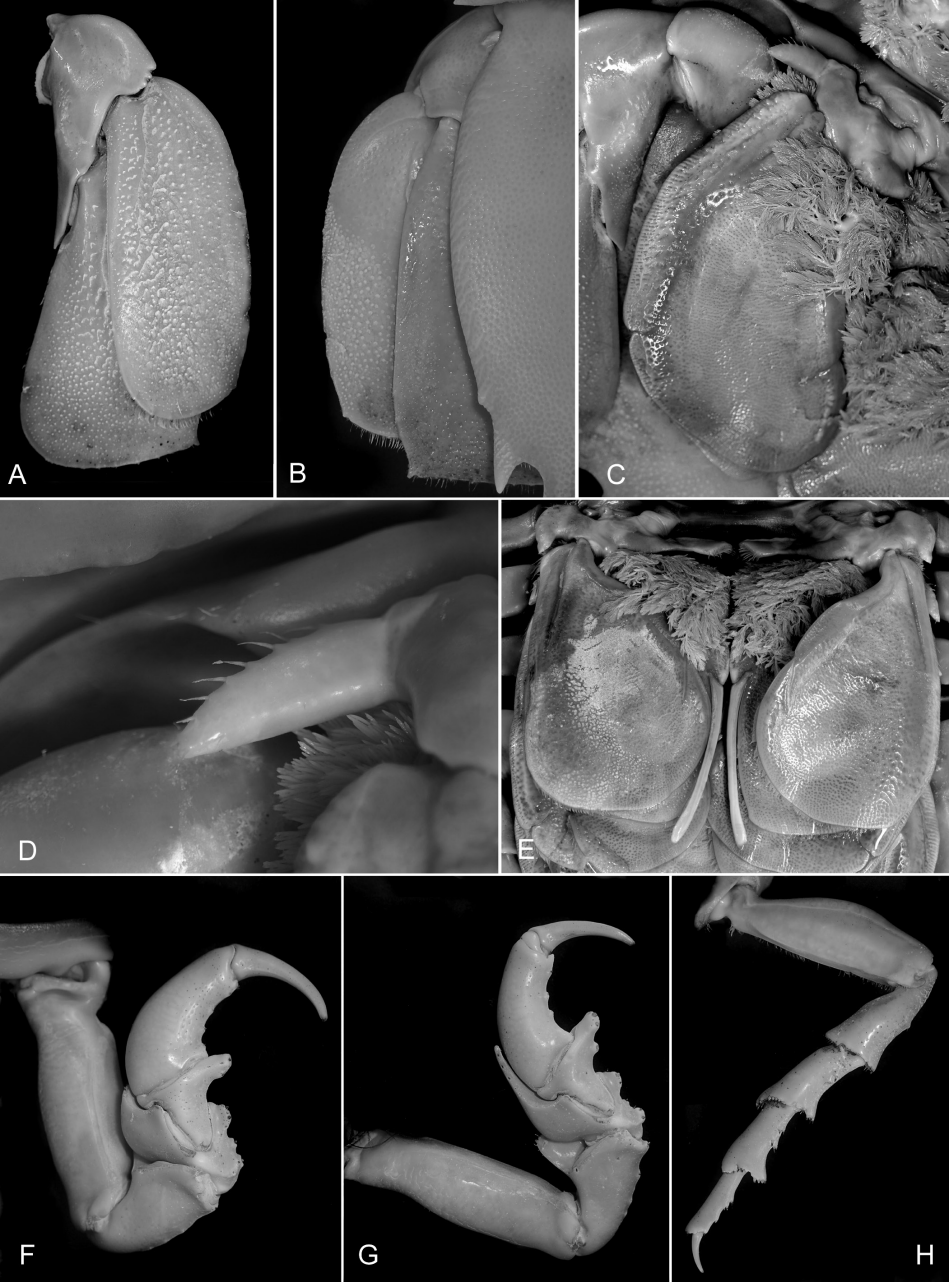
*Bathynomus propinquus* Richardson, 1910, male (320 mm), Verde Island Passage, Philippines, MNHN-IU-2013.6418 (IS.2290): **A, B**. Left uropod, ventral view and dorsal view, respectively; **C**. Right pleopod 5; **D**. Right pleopod 5 exite; **E**. Right and left pleopod 2; **F**. Right pereopod 1; **G**. Right pereopod 2; **H**. Right pereopod 7.

**Figure 7.**
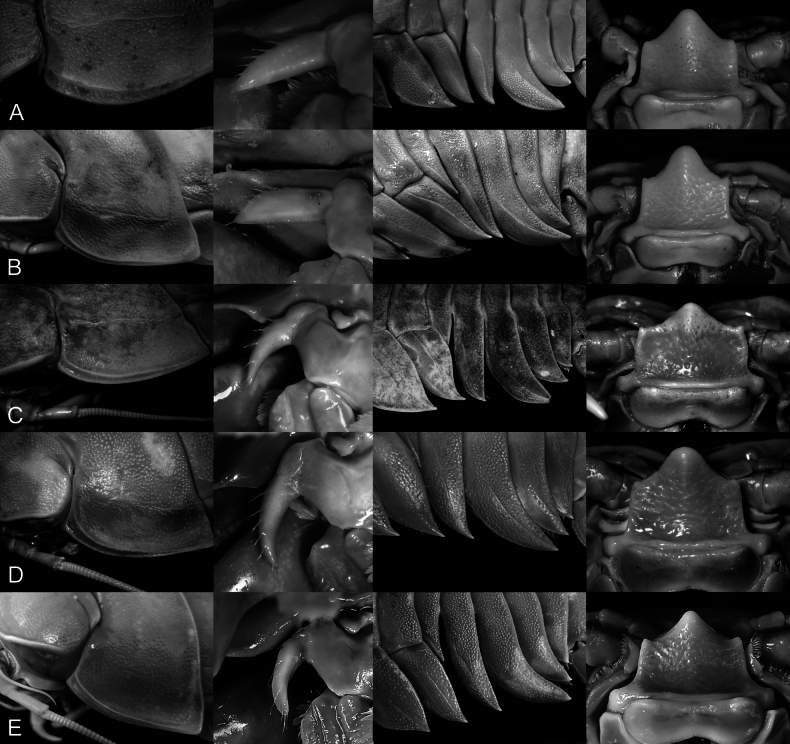
*Bathynomus* spp., comparative diagnostic features, left to right: cephalon and pereonite 1 lateral surface showing marginal carinae; right pleopod 5 exite; pereonites 6–7 and pleonites 1–5, left lateral view; clypeus. **A**. *B. propinquus* Richardson, 1910, male (320 mm), Verde Island Passage, Philippines, MNHN-IU-2013.6418 (IS.2290); **B**. *B. kensleyi* Lowry & Dempsey, 2006, male holotype (277 mm), Queensland, Australia; **C**. *B. vaderi*, male (305 mm), Vietnam, ZRC 2024.0180; **D**. *B. jamesi* Kou, Chen & Li Kou in Chen et al., 2017, male (335 mm), Vietnam, ZRC 2024.0118; **E**. *B. wilsoni* Ahyong, 2025, female holotype (215 mm), Sulu Sea, Philippines, AM P42711.

An additional potentially diagnostic morphological character in *Bathynomus* identified here is the articulated setose lobe on the outer margin of the protopod of the pleopods, identified here as an exite (fide Boxshall and Jaume 2009; Boxshall 2013). Perhaps relevant here is that the monotypic western Indian Ocean genus *Parabathynomus* Barnard, 1924, the only other cirolanid to have pleopodal branchiae, has an epipod on pleopods 1–5 (Kensley 1978). With epipods being a type of exite (i.e. having a respiratory function), the pleopodal epipods of *Parabathynomus* may be homologues of the corresponding exites in *Bathynomus*. At present, however, we retain the more general terminology for the exite in *Bathynomus* given its lobate rather than laminar form (typical of respiratory structures) and the current absence of functional data.

In *Bathynomus*, the shape of the exite on pleopod 5 (Figs 2I, 6D, 7) appears to be diagnostic. The pleopod 5 exite in *Bathynomus*, albeit weakly sclerotised and potentially subject to the vagaries of preservation, appears to be stable in form between juveniles and adults as observed in the series of *B. kensleyi* examined here. In *B. jamesi*, *B. vaderi* and *B. wilsoni*, the pleopod 5 exite is distinctly curved (Fig. 7C–E), but in *B. kensleyi* and the adult of *B. propinquus*, it is linear (Figs 6D, 7A, 7B). Unfortunately, the pleopod 5 exite in the juvenile holotype of *B. propinquus* (Fig. 2I) is difficult to interpret, being soft and having been crushed during preservation. That said, the exite in the holotype still more closely resembles the condition in the adult specimen of *B. propinquus* (MNHN-IU-2013.6418) than in *B. jamesi* sensu stricto.

Recent discussions of the taxonomic distinctions between *B. jamesi* and newly described species (*B. vaderi* and *B. wilsoni*) were based on a composite concept of *B. jamesi* (i.e., inclusive of specimens of both *B. jamesi* sensu stricto and what is now known to be *B. propinquus*). Our recognition of *B. propinquus* as a valid species, however, warrants reappraisal of the diagnostic differences between these aforementioned species as well as *B. kensleyi* from Australia – we refer to this group as the *B. kensleyi* group, being the group of Indo-West Pacific supergiants having upturned pleotelson spines in adults (including *B. lowryi* from the Andaman Sea). To this end, a key to the species of the *B. kensleyi* group is given below.

### Key to adults of the *Bathynomus kensleyi* group

**Table:** 

1	Uropodal exopod setal row short, 67% of lateral exopod margin	***B. lowryi* [Andaman Sea; 690 m]**
–	Uropodal exopod setal row long, 80% or more of lateral exopod margin	**2**
2	When pleon straightened, pleonites 3–5 pleural apices extending posteriorly to same level (or pleuron 4 overeaching pleuron 5) (Fig. 7B, E)	**3**
–	When pleon straightened, pleuron 3 apex not reaching that of pleuron 4, and neither reaching posteriorly to apex of pleuron 5 (Fig. 7C–E)	**4**
3	Uropodal endopod length 2.4–2.5 × width. Pleopod 5 exite linear (Fig. 7C)	***B. kensleyi* [Coral Sea, Australia; 700–1000 m]**
–	Uropodal endopod length 2.1–2.2 × width. Pleopod 5 exite distinctly curved (Fig. 7E)	***B. wilsoni* [Sulu Sea, Philippines; 2150–2500 m]**
4	Length of lateral cephalic incisions about 0.2 × distance between incisions. Dorsal surface of pleotelson in adults pitted but not granular. Pleopod 5 exite linear (Fig. 7A)	***B. propinquus* [Verde Island Passage, central western Philippines; 300–772 m]**
–	Length of lateral cephalic incisions ≥ 0.3 × width between incisions. Dorsal surface of pleotelson in adults pitted and granular. Pleopod 5 exite distinctly curved (Fig. 7C–E)	**5**
5	Clypeus rectangular portion, twice as wide as long or wider (Fig. 7C). Uropodal endopod length 2.5–2.6 × width	***B. vaderi* [northern South China Sea; outer shelf, depth not recorded]**
–	Clypeus rectangular portion, less than twice as wide as long (Fig. 7D). Uropodal endopod length 2.2–2.3 × width	***B. jamesi* [northern South China Sea; 420–898 m]**

### Taxonomy

**Cirolanidae Dana, 1852**

***Bathynomus* A. Milne-Edwards, 1879**

#### Bathynomus propinquus

Taxon classificationAnimaliaIsopodaCirolanidae

Richardson, 1910

21589092-B14E-5E80-AF37-10BE8C3A702A

Figs 1, 2, 3, 4, 5, 6, 7

Bathynomus propinquus Richardson, 1910: 4, fig. 2. — Nierstrasz 1931: 162. — Holthuis and Mikulka 1972: 587–588. — Shih 1972: 31. — Griffin 1975: 103. — Lemos De Castro 1978: 42. — Sekiguchi et al. 1982: 499. — Bruce 1986: 127, fig. 87L–O. — Cocke 1987: 24–29, fig. 5. — Soong 1992: 291, 294. — Magalhães and Young 2003: 221, 227, 238. — Lowry and Dempsey 2006: 164, 168. — Bruce and Shimomura 2019: Table 1.
Bathynomus jamesi . — Ahyong 2025: fig. 8B. — Ng et al. 2025: 291, figs 2A, 9E–H, 10B.
Bathynomus cf. jamesi . — Ng et al. 2026: 187, 188, 197.
NOT Bathynomus propinquus. — Monod 1973: 129, figs 54, 55 [= B. richeri Lowry & Dempsey, 2006].

##### Type material.

***Holotype***: • USNM 40909, juvenile male (88 mm), Verde Island Passage, southern Luzon, Philippines, 13°42'05"N, 120°30'45"E, 422 fathoms (772 m), R/V Albatross sta. D 5284, dredge, 20 July 1908.

**Table 1.** Pairwise distance (%) based on the Kimura 2-parameter (K2P) model of mitochondrial COI (657 bp) with 16S rDNA (531 bp) sequences among species of *Bathynomus* and the outgroup. Numbers in the square brackets are sample size. **$** one specimen with 16S rDNA sequence only (PZ048046). **#** specimen with COI sequence only (*B. jamesi*: PZ044771, *B. kensleyi*: OQ860753, *B. wilsoni*: PZ567722). Except for sequences of *B. wilsoni* and *B. raksasa* newly generated herein, see Ng et al. (2026) for GenBank accession numbers and specimen voucher details.

	**1**	**2^$^**	**3**	**4**	**5^#^**	**6**	**7^#^**	**8**	**9**	**10^#^**	**11**	**12**
**1- *B. affinis* (Philippines)**	*											
**2- *B.* sp (Indonesia)[2]^$^**	7.2–10.8	0										
**3- *B. doederleinii***	12.4	11.8–12.8	*									
**4- *B. giganteus***	20.2	18.2–19.9	19.3	*								
**5- *B. jamesi* [4]^#^**	20.8–25.6	17.7–28.1	21.3–27.2	9.0–13.2	0–0.5							
**6- *B. kapala***	20.9	17.7–21.0	20.8	17.4	17.3–20.4	*						
**7- *B. kensleyi* [4]^#^**	21.6–28.6	18.6–29.2	19.8–30.1	10.6–15.4	8.7–12.0	15.6–20.7	0–0.5					
**8- *B. vaderi* [7]**	21.2–21.8	20.1–22.1	21.5–21.7	11.7–12.3	12.0–17.7	19.4–20.1	12.3–16.8	0–0.4				
**9- *B. propinquus***	20.5	19.3–22.1	22.6	11.3	9.0–10.8	17.3	3.5–4.6	13.8–14.7	*			
**10- *B. wilsoni*^#^**	29.5	28.3	28.2	17.4	13.5–14.1	22.7	6.7–7.5	18.5–18.7	4.0	*		
**11- *B. raksasa***	20.0	17.6–20.3	21.7	9.2	7.1–9.4	20.1	3.3–4.4	11.9–12.7	1.9	5.1	*	
**12- *B. yucatanensis***	20.7	17.9–20.9	18.7	5.2	9.4–14.1	16.7	9.4–16.0	13.5–13.7	10.9	18.9	8.8	*
**13- *Excirolana hirsuticauda***	48.6	47.3–59.6	49.9	52.3	43.5–57.0	48.7	47.0–53.7	49.4–50.6	54.6	48.6	53.1	53.0

##### Other material examined.

• MNHN-IU-2013.6418 (IS.2290), 1 male (320 mm), Verde Island Passage, southern Luzon, Philippines, 13°51'N, 120°30'E, 300–330 m, MUSORSTOM 2 sta. CP75, STA.357, 25 March 1976.

##### Diagnosis of adult.

Cephalon anteromedian surface without shallow, irregularly ridged sulcus; cephalic ridge above eyes discontinuous; lateral incisions demarcating maxillipedal somite extending inward from posterolateral margin for length equivalent to about 0.2 distance between left and right incisions. Pereonite 1 with upper marginal carina meeting anterior margin at distinct, slightly obtuse angle. Pleon, when straightened, with pleonites 3–5 pleural apices with that of pleuron 3 not reaching that of pleuron 4, and neither reaching posteriorly to apex of pleuron 5. Clypeus subpentagonal, lateral margins subparallel, distal margins concave, apex bluntly rounded. Pleopod 5 exite linear. Pleotelson dorsal outline (excluding spines) subtrapezoidal, surface pitted but not granular; posterior margin with 11 or more slender spines, circular–ovate in cross-section, upwardly curved, central spine apically simple. Uropodal exopod length 2.4 × width, setal fringe continuous (length 90%); endopod triangular, distolateral corner approximately obtuse, apex produced to short point.

##### Description.

Body longitudinally ovate, widest at pereonite 4, body length 2.5 × as long as pereonite 4 width (excluding coxae), 2.0–[2.2] including coxae (Figs 1A, 4A); surface densely punctate, without sculpture or granulation. Cephalon width half width of pereonite 1 (including coxae); ridge above eyes discontinuous (Figs 2A, 2E, 5A, 5B); anterior surface pitted and with fine, short, irregular striae, and narrow smooth, arcuate band, neither sulcate nor bordered by irregular ridges (Figs 2A, 5B); maxillipedal somite marked by deep, wide posterolateral incision extending inward from lateral margin, incision length equivalent to 0.3 distance between left and right incisions (Figs 2E, 5A, 5B). Frontal lamina triangular, wider than long, margins weakly carinate (Figs 2A, 5B). Clypeus subpentagonal, lateral margins convergent in juvenile holotype (Fig. 2B), subparallel in adult (Figs 5D, 7A), distal margins concave, apex blunt, narrowly rounded.

Antennular peduncle (Figs 2A, 3B, 5A–D) 4-articulate, reaching almost to midlength of antennal peduncle article 4. Antennal peduncle (Figs 2A, 3B, 5A–D) 5-articulate; article 5 longest, about 1.3 × longer than article 4; flagellum extending to within pereonite 4 in holotype, to within pereonite 2 in adult.

Mandible incisor molar process occlusal margin evenly dentate; palp not reaching incisor, article 1 half length of article 2; article 2 lateral margin setose on distal half; article 3 arcuate, lateral margin setose, length 0.4 × length of article 2. Maxillule lateral lobe occlusal margin with 11 corneous, simple, robust setae in U-shaped row; mesial lobe with 4 robust, circumplumose setae. Maxilla lateral lobe occlusal margin with 9 long simple setae, followed by 6 short simple setae; middle lobe occlusal margin with 8 long simple setae followed by row of shorter simple setae; mesial lobe with fringe of plumose setae of varying length. Maxilliped endite with [4] or 5 coupling hooks.

Pereopods 1–5 coxal plates (Figs 1C, 4C) without fine central ridge, apically rounded on 1–3, bluntly angular on 4, acute on 5, coxa anterior margin gently convex to straight in dorsal view on pereopod 1, gently convex on coxae 2–5.

Pereopod 1 (Fig. 6F) basis length about 3.6 × width. Ischium half as long as basis; with row of 3 or 4 minute robust setae along posterior margin and 3 or 4 robust setae on posterodistal margin. Merus with 3 or 4 robust setae on anterodistal angle; inferior margin with proximal group of 3 or 4 robust setae and distal group of 3 or 4 robust setae. Carpus inferior distal lobe with 2 or 3 robust setae. Propodus length 2.3–[2.5] × width; posterior (occlusal) margin with 4 or [5] robust setae. Dactylus falcate, two-thirds propodus length.

Pereopod 2 (Fig. 6G) basis length about 3.5 × width. Ischium half as long as basis; with row of 3 robust setae along posterior margin and 3 robust setae on posterodistal margin. Merus anterodistal angle produced to midlength of propodus, lined with scattered robust setae; inferior margin with 3 or 4 robust setae in proximal cluster and distal row of 2 or 3 robust setae. Carpus inferior distal lobe with 2 robust setae and 0 or 1 robust seta near distal quarter. Propodus length 2.9 × width; posterior (occlusal) margin with 3 robust setae. Dactylus falcate, two-thirds propodus length.

Pereopod 6 coxal plate (Figs 1C, 2C, 4C, 5E, 7A) with fine central ridge, apex narrowly acute, pointed.

Pereopod 7 (Fig. 6H) coxal plate distally narrowed, gently curved posteriorly, dorsal margin gently sinuous, ventral margin convex, apex narrowly acute, pointed; outer surface with fine central ridge. Basis length 3.4 × greatest width; inferior margin convex; superior margin (flexor) setose. Ischium length 0.6 × basis length, inferior margin with 1 or [2] robust setae near midlength; superior distal angle with 7 or 8 robust setae; inferior distal angle with 8–10 robust setae. Merus length 0.9 × ischium length; inferior margin with 2 robust seta near midlength, superior distal angle with 10 or 11 robust setae; inferior distal angle with 6–9 robust setae. Carpus as long as ischium, inferior margin with 2 (holotype) or 4–6 (adult) robust setae near midlength (as 2+2 or 3+3); superior distal angle with numerous robust setae encircling distal margin. Propodus two-thirds as long as ischium, length 4.4 × width; inferior margin with 2 (holotype) or 3 (adults) pairs of robust setae and single seta, superior distal angle lined with robust seta, inferior distal angle with 5 robust setae. Dactylus 0.5 × propodus length.

Pleonites 1–5 combined length along midline [21]–27% body length (Figs 1A, 4A). Pleon, when straightened, with that of pleuron 3 not reaching that of pleuron 4, and neither reaching posteriorly to apex of pleuron 5 (Figs 1A, 2C, 4A, 5E, 7A); pleura 1–3 surface each with fine central ridge, 4 and 5 without ridge. Penial processes as flat lobes, ovate, distally subtruncate, length twice width, separated by 0.18 × width of sternite 7 (Fig. 1B).

Pleopod 1 (Figs 1B, 4B) exopod almost twice as long as wide, widest slightly distal to midlength, lateral margin almost straight, mesial and distal margins convex; endopod subquadrate, with obtuse triangular lobe on lateral margin, length about 1.8 × width (excluding lobe).

Pleopod 2 (Fig. 6E) exopod subtriangular, distal margin broadly rounded, slightly shorter than pleopod 1 exopod; endopod subquadrate, with obtuse triangular lobe on lateral margin, length about 1.6 × width (excluding lobe), as long as pleopod 1 endopod; adult male with appendix masculina slender, extending slightly beyond apices of exopod and endopod.

Pleopods 3 and 4 with shape and proportions similar to pleopod 2.

Pleopod 5 (Fig. 6C) exopod subovate, twice as long as wide, widest slightly distal to midlength, lateral margin strongly convex, mesial margin weakly convex; endopod spatulate, mesial margin sinuous, distal margin rounded, lateral margin strongly convex, with obtuse triangular lobe on lateral margin, length about twice width (excluding lobe); exite stout, linear, anterior margin broadly convex, with 6–8 simple setae.

Pleotelson length 0.7–0.8 × greatest width, dorsal outline (excluding spines) subtrapezoid, widest at anterior 0.3 (Figs 1A, 2D, 4A, 5G); dorsal surface smooth (minutely pitted), evenly convex in cross section, with inconspicuous (scarcely discernible, even when surface is dried) longitudinal median carina, straight in lateral view. Posterior margin with 12 or [13] spines of which 11 are prominent, holotype with lateralmost spine on either side represented by small notch (Fig. 2H), adult with low tubercle on left side (Fig. 5G); remaining spines with flattened, triangular bases but broken apices in holotype, in adult, spines slender, prominent, upwardly curved, circular-ovate in cross-section (Figs 4A, 5F–H), with sparse row of setae between spines, central spine simple in adult (broken in holotype).

Uropods not reaching end of pleotelson (Figs 1, 2). Peduncle with 2 or 3 small robust setae on anteroventrolateral margin (Figs 2G, 6A). Exopod (Figs 2G, 2H, 6A, 6B) length 2.4–[3.2]× width; lateral margin broadly convex, with 9–[14] robust setae along margin, setal fringe continuous (length 90%); mesial margin straight; distomedial corner convex with 4 or 5 robust setae, distolateral angular, weakly produced. Endopod (Figs 2G, 2H, 6A, 6B) triangular, length twice width; lateral margin broadly convex, with 4–[6] robust setae; mesial margin straight, distomesial corner rounded, produced; distal margin straight with 10–[14] robust setae; distolateral corner obtuse, apex produced to short point.

##### Remarks.

*Bathynomus propinquus* belongs to the group of supergiants sharing upcurved posterior pleotelson spines in adults, which, in the Indo-West Pacific includes *B. jamesi*, *B. kensleyi*, *B. lowryi*, *B. vaderi* and *B. wilsoni*. *Bathynomus propinquus* closely resembles *B. jamesi* in sharing a similar alignment of the pleonite 3–5 pleura in which the successive pleural apices reach further posteriorly when the pleon is straightened such that pleuron 3 is shorter than pleuron 4 and neither reach posteriorly to or beyond pleuron 5 (Figs 1A, 2C, 4A, 5E, 7A), the apex of the pereonite 7 coxal plate does not taper to form a slender curved posteriorly directed spine (as in *B. vaderi*; Fig. 7A versus 7C), and a similarly elongate appendix masculina that extends slightly beyond the corresponding endopod lamina (Fig. 6E). *Bathynomus propinquus*, however, is readily distinguished from *B. jamesi* by the shorter lateral incisions in the cephalon (length about 0.2 × distance between incisions versus 0.3 × or greater), the distinctly angular junction between the upper marginal carina and anterior margin of pereonite 2 (Figs 2F, 5C, 7A) (versus curved junction; Fig. 7B, D, E), absence of dorsal granulation on the upper surface of the pleon and pleotelson in adults (versus densely granulate), and linear pleopod 5 exite (Figs 6D, 7A) (versus distinctly curved; Fig. 7C–E)). Recognition that some material previously thought to represent *B. jamesi* is referable to *B. propinquus*, along with additional material of the former, provides opportunity to refine adult distinguishing characters. In particular, *B. propinquus* has a proportionally more slender uropodal exopod in adults, with length:width ratio 2.4 (Fig. 6A) (like *B. kensleyi*, 2.4–2.5), compared with 2.2–2.3 in adult *B. jamesi* (overlapping with *B. wilsoni* at 2.1–2.2). *Bathynomus propinquus* differs from *B. vaderi* in most of the same features as for *B. jamesi*, but further differs in the more elongate appendix masculina (extending slightly beyond versus not beyond the corresponding endopod lamina; Fig. 6E, Ng et al. 2025: fig. 10A, B), form of the pereopod 7 coxa (apex acute but not tapered to slender curved tip; Figs 2C, 5E, 7A, 7C), and proportionally narrower clypeus (Figs 5D, 7A) (versus widely rectangular; Fig. 7C). *Bathynomus propinquus* is readily distinguished from *B. kensleyi*, *B. lowryi* and *B. wilsoni* by the alignment of the pleonite 3–5 pleura, which, in the latter three species, all reach posteriorly to the same level or have pleuron 3 overreaching pleuron 5 (Fig. 7B, E).

Based on mitochondrial COI+16S sequences, *B. propinquus* forms a well-supported clade together with *B. kensleyi* and *B. wilsoni* (Fig. 3A), rather than with other South China Sea species such as *B. jamesi*, with which Verde Passage material was formerly misidentified. *Bathynomus propinquus*, *B. kensleyi* and *B. wilsoni* are all dorsally smoother than *B. jamesi*, with the non-granulate rather than prominently granulate dorsum in adults. The three species are also genetically less divergent from each other (mean pairwise divergence 3.5–7.5%) than to other analysed congeners (≥ 8.7%) (Table 1).

The close phylogenetic relationship between *B. propinquus* and *B. wilsoni* is biogeographically intuitive, although the latter appears to be morphologically more similar to the Australian *B. kensleyi* sharing more elongated lateral cephalic incisions and similar alignment of the pleonite 3–5 pleura. Conversely, *B. propinquus* and *B. kensleyi* share similar length:width proportions (2.4–2.5 versus 2.1–2.2) of the uropodal exopod and the linear rather than curved pleopod 5 exite.

With the present clarification of the distinctions between *B. jamesi* and *B. propinquus*, it is apparent that the former occurs on the northern continental slope margins of the South China Sea (Vietnam to Hainan and Dongsha) whereas the latter is a species of the southeastern slope margins; none are yet recorded from the bathyal or abyssal depths of the central South China Sea.

##### Distribution.

Currently known only from the Verde Island Passage, central-western Philippines; 300–772 m.

#### Bathynomus wilsoni

Taxon classificationAnimaliaIsopodaCirolanidae

Ahyong, 2025

E50EAF9C-037C-5937-A2F8-5F56D3A4862B

Fig. 8

Bathynomus kensleyi . — Lowry and Dempsey 2006: 184 [Sulu Sea specimens only; not B. kensleyi Lowry & Dempsey, 2006]. — Richer de Forges et al. 2013: fig. 7.
Bathynomus sp. — Richer de Forges et al. 2009: tab. 2.
Bathynomus wilsoni Ahyong, 2025: 170–181, figs 1–7, 8A. — Ng et al. 2026: 194.

##### Material examined.

• ZRC 2018.1076, 1 juvenile female (TL 145 mm), Sulu Sea, south of Siaton, 8°51.3'N, 122°58.9'E, 2150–2387 m, sandy bottom, PANGLAO 2005, CP2387, beam trawl, 29 May 2005; • AM P42711, female holotype (215 mm), Sulu Trough, Sulu Sea, Philippines, 8°58.4'N, 121°21.1'E to 8°58.4'N, 121°21.2'E, 2500 m, thermally insulated tube trap, PAPATUA Expedition Leg 08, PPTU08WT, RV Thomas Washington, coll. A.A. Yayanos, 10–11 April 1986; • ZRC 2025.0098, male paratype (212 mm), collected together with holotype.

**Figure 8.**
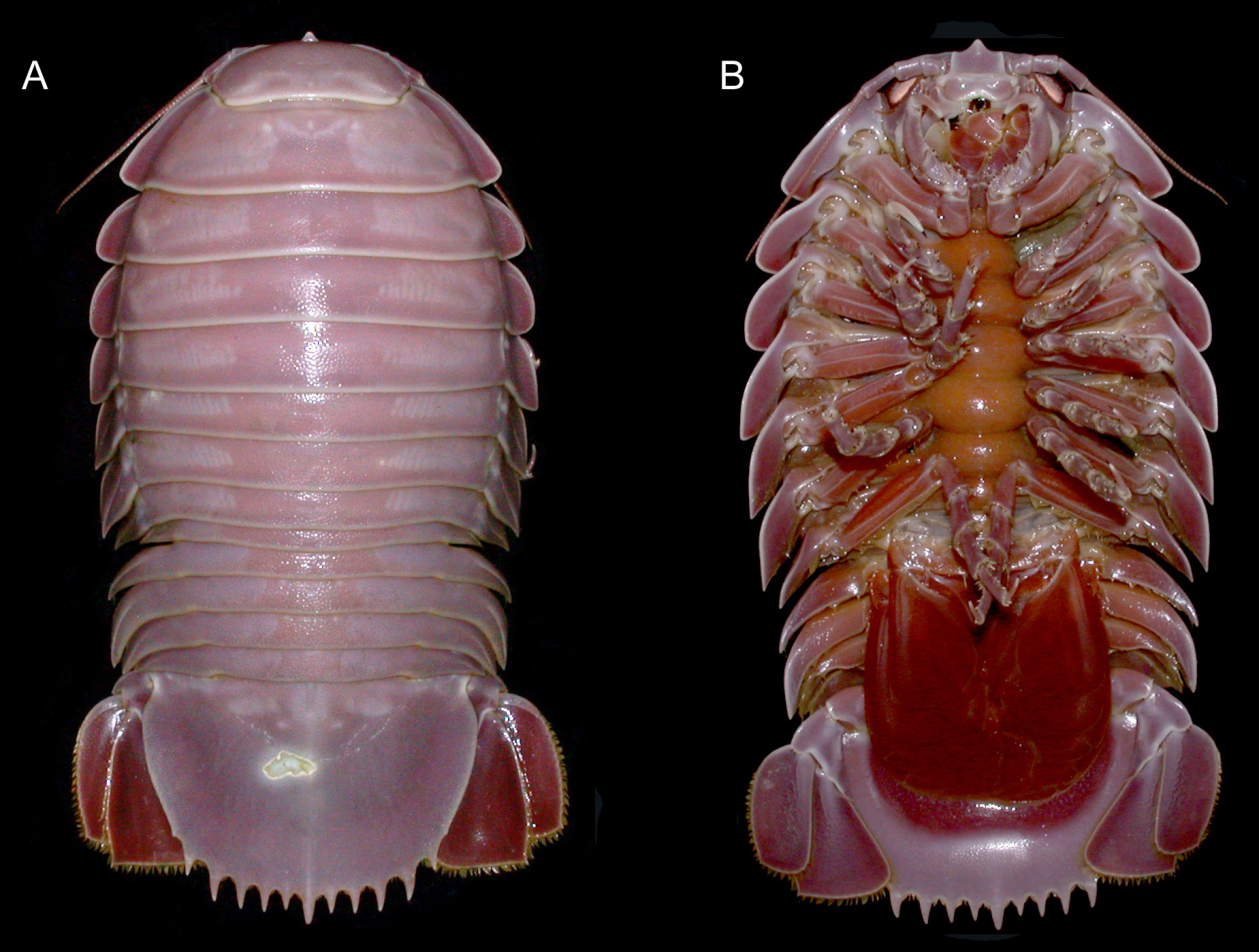
*Bathynomus wilsoni* Ahyong, 2025, colour in life, juvenile female (145 mm), Sulu Sea, Philippines, ZRC 2018.1076: **A**. Dorsal habitus; **B**. Ventral habitus. Photograph: T.Y. Chan.

##### Remarks.

One specimen of a species of *Bathynomus* was collected from the Philippines during PANGLAO 2005, an anterior photograph of which, was provided by Richer de Forges et al. (2013: fig. 7) as *B. kensleyi* but was not available for study until recently. We have since had opportunity to examine the specimen (Fig. 8), a juvenile female of a species of supergiant collected from the Sulu Sea at 2150–2387 m. The specimen best corresponds to *B. wilsoni*, described from the Sulu Sea at 2500 m depth, agreeing in most respects with the type material including the elongated lateral cephalic incisions (length about 0.3 × distance between incisions), similar alignment of the pereonite 3–5 pleura (which reach posteriorly to the same level), and similar clypeal form; there is a row of 11 prominent pleotelson spines flanked on either side by a tiny or incipient spine. The juvenile female reported here has well developed pereopods 7 but lacks oostegites, corresponding to subadult stage II of Soong and Mok (1994); the mesial margins of coxae 1–3 have a shallow crescent-shaped groove, which may represent the eruption sites of the oostegites.

The juvenile differs from adults of *B. wilsoni* in aspects attributable to its immaturity: posterior pleotelson spines are straight rather than upcurved, and the central spine is undivided (although the apex appears to have possibly been damaged or worn); the uropodal exopod lateral margin is yet to attain the strong convexity of adults, so the article is more slender, with a length: width ratio of 2.4 versus 2.1–2.2; the pleotelson dorsal outline is subpolygonal rather than rounded, and the low median ridge is weakly convex rather than straight in lateral view.

The present specimen is the third known of *B. wilsoni* and extends its known bathymetric range into slightly shallower depths. Nevertheless, with a bathymetric range of 2150–2500 m, *B. wilsoni* is conspicuous as the only known bathyal species of the genus in the Indo-West Pacific. Only the western Atlantic *B. giganteus* is also known from bathyal depths (Holthuis and Mikulka 1972). All other species of *Bathynomus* occur at slope depths shallower than 1000 m, except for *B. keablei* Lowry & Dempsey, 2006, at 1016–1353 m from the Bay of Bengal (Annandale 1909; Lowry and Dempsey 2006) and *B. raksasa* with the deepest record at 1259 m (holotype), Sunda Strait, Indonesia (Sidabalok et al. 2020).

##### Colour in life.

The young specimen of *B. wilsoni* agrees well with that reported in Ahyong (2025: fig. 7): dorsal surface of body pale mauve-pink with cream-coloured somite posterior margins; ventrally with pereonal sternites dull orange-brown; pereopods, pleopods and uropods dark maroon (Fig. 8).

##### Distribution.

Known only from the Sulu Sea, southern Philippines; 2150–2500 m.

## Supplementary Material

XML Treatment for Bathynomus propinquus

XML Treatment for Bathynomus wilsoni

## Additional information

### Artificial Intelligence (AI) use

The authors accept full responsibility for the content of the manuscript, including the disclosure of any use of AI.

No AI tools were used in the preparation of this manuscript.

### Author ORCIDs

S.T. Ahyong https://orcid.org/0000-0002-2820-4158

C.M. Sidabalok https://orcid.org/0000-0002-4325-0558

C.-H. Yang https://orcid.org/0000-0002-4594-3622

P.K.L. Ng https://orcid.org/0000-0001-5946-0608

### Data availability

All of the data that support the findings of this study are available in the main text.
